# Evaluation of diagnostic efficiency of the new enzyme immunoassay for detection of IgM antibodies to Hepatitis B core antigen

**DOI:** 10.1186/1471-2334-14-S2-P85

**Published:** 2014-05-23

**Authors:** OF Fedorova, NM Khodak, NA Bugrova, VF Puzyrev, AN Burkov, TI Ulanova

**Affiliations:** 1 RPC «Diagnostic Systems», Nizhny Novgorod, Russia; 2 DSI S.R.L. Saronno, Italy

## Introduction

Recently, many studies are available about the detection of viral DNA in sera containing IgM to HBcAg in the absence of HBsAg, which lead to increase the risk of transmission of HBV, what is especially important for blood transfusion and the use of blood products. In this regard, it is important to use highly sensitive ELISA test for detection of IgM against HBcAg which could help to eliminate the risk of transmission of HBV, especially during the «serological window».

## Aim

To assess the diagnostic efficiency of the new high sensitive test for the IgM to the core antigen of HBV detection with the aim to use it for early diagnostic Hepatitis B virus.

## Materials and methods

Analytical sensitivity of the kit was evaluated using standard of Paul-Ehrlich Institut - HBc-Referenzserum-IgM 84 (IgM anti-HBc). Diagnostic sensitivity of the kit was evaluated using samples of Anti-HBc IgM Performance Panel PHE203 (BBI), four seroconversion panels produced by ZeptoMetrix and BBI (USA) and 264 positive samples from patients with HBV at various stages. Diagnostic specificity was evaluated by testing different categories of the population: primary donors (n=1056), clinical patients (n=386), potentially cross-reacting samples (n=509). Diagnostic efficiency of the kit was evaluated in comparison with the reference kits with the analytical sensitivity of 25-50 U/ml PEI.

## Results

Analytical sensitivity of the new test is ≤10 U/ml PEI. The seroconversion panels study has found that the kit detects 56 positive samples out of 92 seroconversion samples, compared with commercial reference assays, which detect 46 samples. The diagnostic sensitivity of the developed assay is 100% (264/264) compare to the 41% (108/264) of the reference test that may be caused by higher sensitivity level of the new test in comparison with the reference test. Diagnostic specificity of the kit is 99.6%.

## Conclusion

High sensitivity and specificity of the new test provide more effective anti-IgM detection which is important for the diagnostic of the current or past infection of HBV.

